# Dietary live microbe intake and its association with Parkinson’s disease in American adults: an NHANES analysis (1999–2018)

**DOI:** 10.3389/fnut.2025.1606922

**Published:** 2025-05-29

**Authors:** He Zou, Tao Zhu, Xiaoshu Chen, Yifei Lu

**Affiliations:** ^1^Department of Cardiology, Wenzhou Third Clinical Institute Affiliated to Wenzhou Medical University, Wenzhou People’s Hospital, Wenzhou, China; ^2^Department of Cardiology, Taizhou Hospital of Zhejiang Province Affiliated to Wenzhou Medical University, Taizhou, China

**Keywords:** NHANES, Parkinson’s disease, dietary live microbe, MedHi, obesity

## Abstract

**Background:**

Diet plays a crucial role in sustaining a healthy body, and microbes have attracted significant scholarly interest in recent years as an essential component of diet. This study aims to explore the association between dietary live microbe intake and the incidence of Parkinson’s Disease (PD) among adults over 40 years old.

**Methods:**

A total of 26,033 subjects in NHANES 1999–2018 were included in this study, comprising 314 patients with PD, which were divided into three groups based on their intake of foods with different levels of microbial content: high, medium, and low. The assessment on subjects’ dietary live microbe intake was conducted through the self-reported questionnaire.

**Results:**

The distribution of subjects based on dietary live microbe intake was as follows: 34.5% had a low intake, 45.4% had a medium intake, and 20.1% had a high intake. A multivariable logistic regression analysis revealed that subjects with high (OR = 0.52, 95% CI: 0.37–0.74) and medium (OR = 0.73, 95% CI: 0.57–0.93) dietary live microbe intake exhibits a reduced prevalence of PD compared to those with low intake. Restricted cubic spline (RCS) analysis indicated a significant linear association between dietary live microbe intake and the prevalence of PD. Furthermore, stratified analyses demonstrate that the association among research variables was more pronounced in subjects without obesity.

**Conclusion:**

This study demonstrates an inverse linear association between dietary live microbe intake and the prevalence of PD.

## Introduction

Parkinson’s disease (PD) manifests as a clinical syndrome typified by symptoms, such as cogwheel rigidity, bradykinesia, postural instability, slow shuffling gait, and resting tremors (1). As the second most prevalent neurodegenerative disorder followed by Alzheimer’s disease, PD constitutes a significant societal concern and poses a critical global health challenge. Projections indicate that the incidence of PD will double over the next 30 years (2–5), with the increasing affected subjects from 6.9 million in 2015 to 14.2 million by the end of 2040 (6). Understanding the risk factors and pathogenesis of PD is crucial for improving the prognosis of those impacted by the disease and their quality of life.

The etiology of PD has been widely recognized as multi-factorial, involving an interplay of environmental and genetic influences (7, 8). Among these environmental determinants, diet has emerged as a significant factor. It is well established that microorganisms are prevalent in various foods consumed, such as natural foods and fermented products (9). These microorganisms influence both the preservation and flavor profile of food and are intricately associated with health conditions (10). Growing evidence has shown that gut microbiota may be the important contributor to central nervous system (CNS) dysfunction (11, 12). Gut microbiota alterations can increase both intestinal and blood–brain barrier permeabilities, leading to a continuous accumulation of gut microbiota-derived molecules and metabolites in brain, thereby promoting neuroinflammation in CNS (13). Gut dysbiosis has been found to be strongly associated with the pathology of PD in all clinical and preclinical studies (14–17). Alterations in microbiome induced microglial activation and neuroinflammation in mice overexpressing *α*-Syn, in turn promoting motor dysfunction of mice (16).

Dietary live microbes see those microorganisms capable of surviving in the gastrointestinal tract and exerting beneficial effects, including probiotic bacteria and other advantageous microorganisms (probiotic candidates) (18). These microbes may prevent the colonization of exogenous pathogenic bacteria within the gastrointestinal tract by promoting the homeostasis of intestinal microbiota and inhibiting harmful bacteria. And metabolites produced by the colonization of microbes in the gastrointestinal tract will also neutralize and inhibit the production of harmful bacterial toxins, reducing local and systemic inflammatory reactions in human body (19). Substantial research has established probiotics contributing to keep healthy by modulating immune function and gut microbiota (20, 21), with observed benefits in various conditions, including digestive disorders, chronic liver disease, chronic kidney disease, Alzheimer’s disease, and PD (22–25). Furthermore, an increased intake of synbiotics and probiotics has been demonstrated to alter gut microbial composition, thereby supporting healthy aging (26–29).

**Figure 1:**
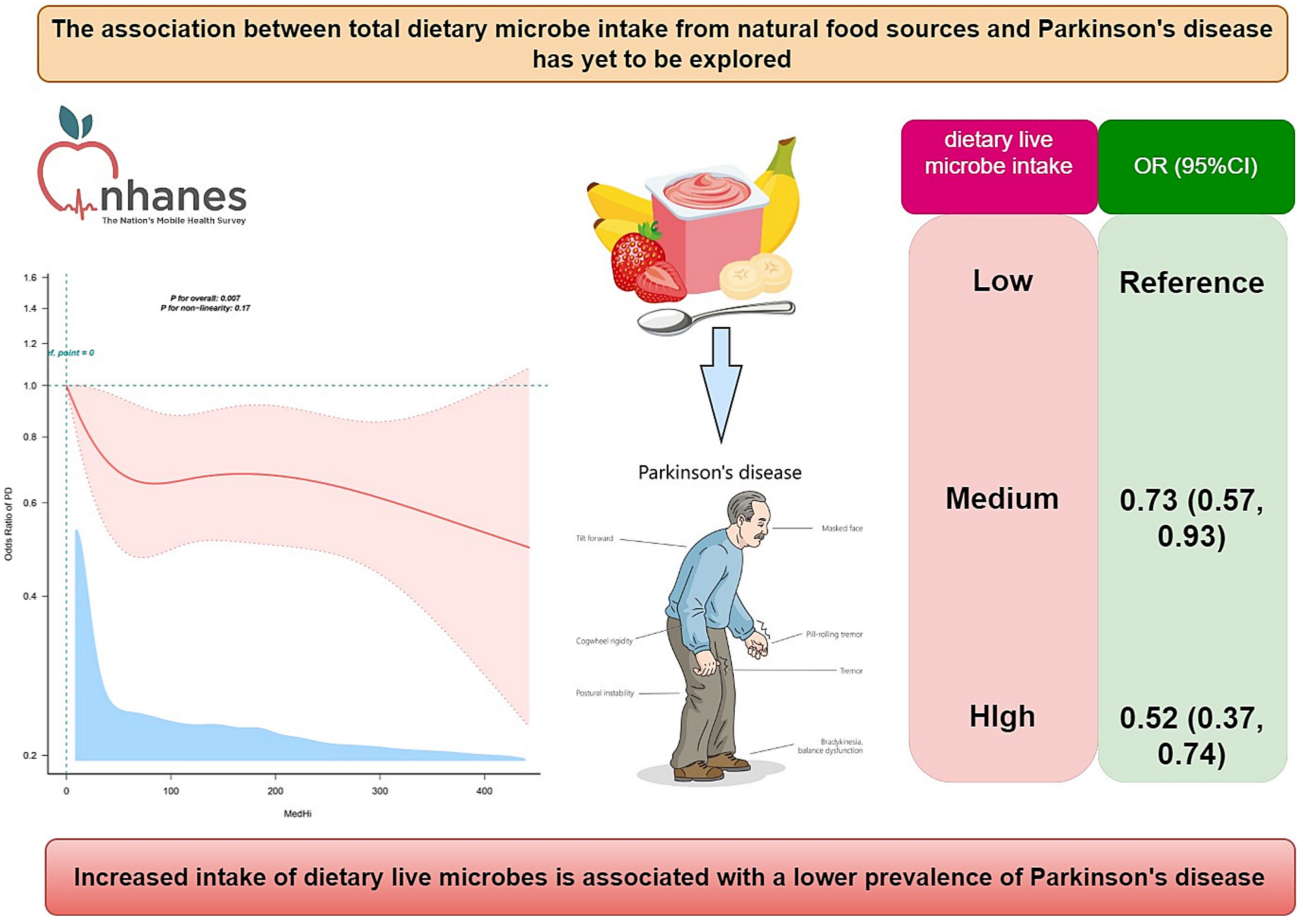
The graphic summary in this study.

In summary, PD has been considered to be associated with the dysbiosis of systemic inflammation and gut microbiota, suggesting that dietary live microbes may serve as a potential target for the management and prevention of PD. While previous studies have predominantly centered on the advantages of probiotic supplements, there is a significant gap in the research on the exploration of the impact of total intake derived from natural foods. Dietary live microbes exist in various foods, including unpeeled fruits and vegetables, as well as fermented dairy products (30). These natural foods are easy to obtain, inexpensive, and can be consumed regularly. Given that consuming dietary live microbes through food sources might provide a comprehensive and sustainable means to enhancing immune function and gut health, it is crucial to explore the association between dietary live microbes and PD. Based on previous studies that estimated dietary live microbe intake based on the viable bacteria per gram of food (31, 32), this study examined the dietary live microbe intake in American adults from NHANES 1999–2018 and explored its association with PD in American adults. We hypothesized that higher intakes of dietary live microbes would be associated with a lower risk of PD (see Figure 1).

## Methods

### Research subjects and design

NHANES, a comprehensive research initiative conducted biennially by NCHS, aims to assess the association among disease prevention, nutrition, and health promotion (33), by taking a series of interviews and physical examinations, encompassing a wide range of sections including dietary, demographic, and laboratory data, as well as various health assessments.

**Figure 2:**
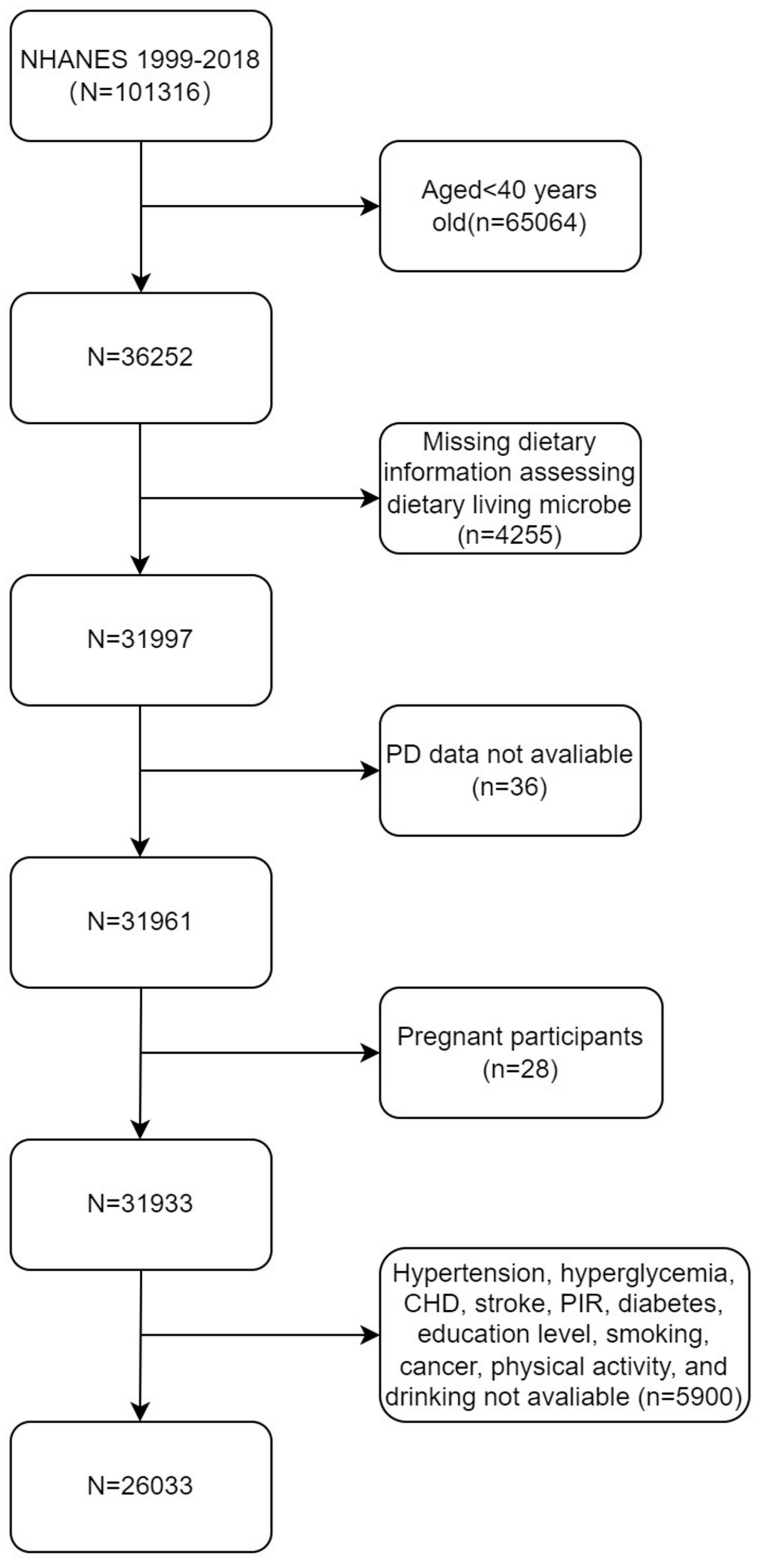
Flowchart of the sample selection from the 1999–2018 NHANES.

In this study, a retrospective analysis was conducted on the data from 10 cycles of the NHANES dataset spanning the period from 1999 to 2018. Subjects aged 40 years old and older (*n* = 36,252) were included. Based on predefined exclusion criteria, subjects with incomplete data for dietary information required to assess dietary live microbe intake (*n* = 4,255) were excluded. Specifically, these subjects lacked the information on the types or quantities of foods consumed, which is essential for estimating microbial content accurately. Additionally, subjects without data on PD status (*n* = 18,610) were excluded, as PD diagnosis was a critical variable for this study. Pregnant females (*n* = 28) were excluded due to the unique physiological and dietary changes during pregnancy, which could confound the association between dietary live microbe intake and PD. Lastly, subjects with missed covariate data (*n* = 5,900), including demographic information, health conditions, or lifestyle factors, were excluded to ensure the completeness and reliability of the data for multivariable analysis. As a result, the final sample comprised 26,033 subjects, as illustrated in Figure 2.

### Assessment on dietary live microbe intake

In this study, the data on dietary intake of live microbes were obtained through the estimation of live bacterial quantities (per gram) conducted by four domain experts (MES, MLM, CH, and RH). These experts evaluated 9,388 food codes across 48 subgroups within the NHANES database (30). The estimation process employed 24-h dietary recall data from the United States Department of Agriculture (USDA) and was cross-referenced with corresponding food codes in the NHANES database, ensuring alignment with the food and nutrient database (31, 32). The microbial content was categorized into three levels: High (>10^7^ CFU/g), medium (10^4^–10^7^ CFU/g), and low (<10^4^ CFU/g). Foods characterized by low microbial content predominantly consist of heat-treated or pasteurized items, including milk, gravies, seafood, prepared meats, and sauces. In contrast, foods with medium microbial content were primarily unpeeled fresh vegetables and fruits, which inherently harbored moderate levels of microorganisms. Additionally, foods with high microbial content encompass unpasteurized fermented products, such as kefir, yogurt, and kimchi, in addition to probiotic supplements.

Subjects were stratified into three distinct groups according to microbial content in the diet: low intake (all consumed foods demonstrated low microbial content), medium intake (any consumed food demonstrated medium but not high microbial content), and high intake (any consumed food demonstrated high microbial content). Moreover, subjects were divided into three categories based on the intake of MedHi foods, which quantified the dietary live microbe intake: Group 1 including those who did not consume any MedHi foods; Group 2 including those whose MedHi food intake was above zero but below the median level; and Group 3 including those whose MedHi food intake was above the median level.

### Diagnosis of PD

In this study, subjects with PD were ascertained by pinpointing those prescribed “Anti-Parkinson Agents” based on the subjects’ self-reported prescription medication usage. Given the limitations of the NHANES dataset concerning available medication codes, the diagnosis of PD was attributed solely to those currently receiving treatment. Consequently, those who did not report anti-Parkinsonian medication were deemed as non-PDs, defining PD aligned with the diagnostic criteria, which has been employed in previous studies (34, 35).

### Covariates

Based on previous studies (36) and variables provided by NHANES, covariates of interest including self-reported health conditions, physical examination results, and sociodemographic variables, were identified as potential confounding factors. Trained researchers collected self-reported health information and sociodemographic variables, including smoking status, total energy intake, past medical history, and alcohol abuse during MEC and family interviews. Sociodemographic variables included education level, age, marital status, gender, PIR, and race. Physical examination results, including blood pressure and BMI, were collected by trained health technicians at MEC. Additionally, the definitions for diabetes, hypertension, and hyperlipidemia were consistent with those previously reported (37). The incidence of stroke, coronary heart disease (CHD), and cancers was determined through self-reported diagnoses. Alcohol abuse was characterized by taking at least 12 alcoholic beverages within 1 year. Smoking was defined by taking a minimum of 100 cigarettes over lifetime. The assessment on probiotic supplement intake was carried out through the dietary supplement questionnaire (30).

### Statistical analysis

The normality of continuous variables was assessed by representing them as either the median with interquartile range or mean ± SD. Through multivariable logistic regression analyses, the association between dietary live microbe intake and PD was explored across three progressively adjusted models: Model 1 (unadjusted), Model 2 (accounted for gender, race, and age) and Model 3 (extensively adjusted for an array of factors, including gender, age, race, BMI, physical activities, diabetes, education level, hypertension, smoking status, CHD, stroke, PIR, total energy intake, cancers, hyperlipidemia, marital status, and alcohol abuse). The heterogeneity between dietary live microbe intake and PD was explored through interactions and subgroup analyses on various variables. In addition, RCS regression analysis was conducted to assess the linearity and explore the dose–response association between dietary live microbe intake and PD, adjusting for covariates included in Model 3. During the sensitivity analyses, multiple logistic regression analyses were performed to further explore the association between dietary live microbe intake and PD, including extra adjustments for the intake of non-food prebiotics and probiotics. The statistical assessments were carried out with Free Statistics software (Version 2.0, China) and R software (Version 4.1.1, Vienna, Austria).

## Results

### Demographic characteristics

Table 1 delineates the baseline characteristics of all subjects, categorized based on the absence or presence of PD. Among the cohort, 314 patients (1.2%) were diagnosed with PD, with the average age of 60 years old, including 13,192 (50.7%) females. In terms of dietary live microbe intake classified by varying microbial content, 34.5% of adults had a low intake, 45.4% had a medium intake, and 20.1% had a high intake. According to the MedHi classification, 34.5, 32.5, and 32.9% of the adults were categorized into groups as G1, G2, and G3, respectively. Subjects with PD were more likely to be older, non-Hispanic White, living alone, with a higher BMI, a lower household income, and a combination of hypertension, CHD, and stroke (*p* < 0.05). The proportion of subjects with a high dietary live microbe intake was significantly lower among PD subjects compared to non-PD subjects (*p* < 0.05). The basic attributes of the subjects, divided into high, medium, and low intake groups, as well as G3, G2, and G1 groups, are shown in Supplementary Tables S1, S2. Notably, the high intake group had a lower prevalence of PD (*p* < 0.05).

**Table 1:** Baseline characteristics of the study participants.

Characteristics	Total (*n* = 26,033)	Non-PD (*N* = 25,719)	PD (*n* = 314)	*p*-value
Age, year	60.0 ± 12.6	60.0 ± 12.6	64.1 ± 13.2	<0.001
Gender, *n* (%)				0.504
Male	12,841 (49.3)	12,692 (49.3)	149 (47.5)	
Female	13,192 (50.7)	13,027 (50.7)	165 (52.5)	
PIR	2.68 ± 1.62	2.68 ± 1.62	2.29 ± 1.55	<0.001
BMI, kg/m^2^	29.3 ± 6.5	29.3 ± 6.5	30.0 ± 6.9	0.044
Race, *n* (%)				<0.001
Mexican American	4,245 (16.3)	4,213 (16.4)	32 (10.2)	
Other Hispanic	2045 (7.9)	2021 (7.9)	24 (7.6)	
Non-Hispanic White	12,816 (49.2)	12,618 (49.1)	198 (63.1)	
Non-Hispanic Black	5,328 (20.5)	5,278 (20.5)	50 (15.9)	
Other Race	1,599 (6.1)	1,589 (6.2)	10 (3.2)	
Physical activity, *n* (%)				0.101
Low	22,049 (84.7)	21,783 (84.7)	266 (90.5)	
High	3,984 (15.3)	3,936 (15.3)	48 (9.5)	
Marital group, *n* (%)				0.002
Married or living with partner	17,337 (66.6)	17,154 (66.7)	183 (58.3)	
Widowed or divorced	6,780 (26.0)	6,685 (26)	95 (30.3)	
Never married	1916 (7.4)	1880 (7.3)	36 (11.5)	
Education level, *n* (%)				0.154
Less than high school	7,673 (29.5)	7,569 (29.4)	104 (33.1)	
High school or above	18,360 (70.5)	18,150 (70.6)	210 (66.9)	
Smoking status, *n* (%)				0.516
Current or ever	13,123 (50.4)	12,959 (50.4)	164 (52.2)	
Never	12,910 (49.6)	12,760 (49.6)	150 (47.8)	
Drinking status, *n* (%)				0.430
Current or ever	17,781 (68.3)	17,573 (68.3)	208 (66.2)	
Never	8,252 (31.7)	8,146 (31.7)	106 (33.8)	
Diabetes, *n* (%)	5,021 (19.3)	4,948 (19.2)	73 (23.2)	0.073
Hypertension, *n* (%)	12,148 (46.7)	11,972 (46.5)	176 (56.1)	<0.001
Hyperlipidemia, *n* (%)	20,017 (76.9)	19,780 (76.9)	237 (75.5)	0.550
CHD, *n* (%)	1,633 (6.3)	1,605 (6.2)	28 (8.9)	0.049
Stroke, *n* (%)	1,317 (5.1)	1,285 (5)	32 (10.2)	<0.001
Cancer, *n* (%)	3,486 (13.4)	3,434 (13.4)	52 (16.6)	0.097
Total energy intake, kcal	1976.6 ± 910.2	1977.7 ± 911.0	1882.7 ± 843.8	0.066
Category of MedHi^a^				<0.001
Low	8,990 (34.5)	8,853 (34.4)	137 (43.6)	
Medium	11,808 (45.4)	11,674 (45.4)	134 (42.7)	
High	5,235 (20.1)	5,192 (20.2)	43 (13.7)	
Category of MedHi^b^				<0.001
G1	8,990 (34.5)	8,853 (34.4)	137 (43.6)	
G2	8,472 (32.5)	8,381 (32.6)	91 (29)	
G3	8,571 (32.9)	8,485 (33)	86 (27.4)	

Values are mean ± SD, median (IQR) or number (%). BMI, body mass index; PIR, poverty-income ratio; CHD, coronary heart disease.

^a^Participants were also classified into three different groups considering the general intake of foods with varying contents of microbe: low (all foods consumed were Low); moderate (any foods consumed were Medium but not High); and high (any foods consumed were High).

^b^Participants were categorized into three groups based on the MedHi consumption to quantify the ingestion of live microbes: G1, consumers without intakes of any MedHi food; G2, those with intakes of MedHi food above zero but below the median level for consumers; G3, those with intakes of MedHi food above the median level for consumers.

### Association between dietary live microbe intake and PD

**Figure 3:**
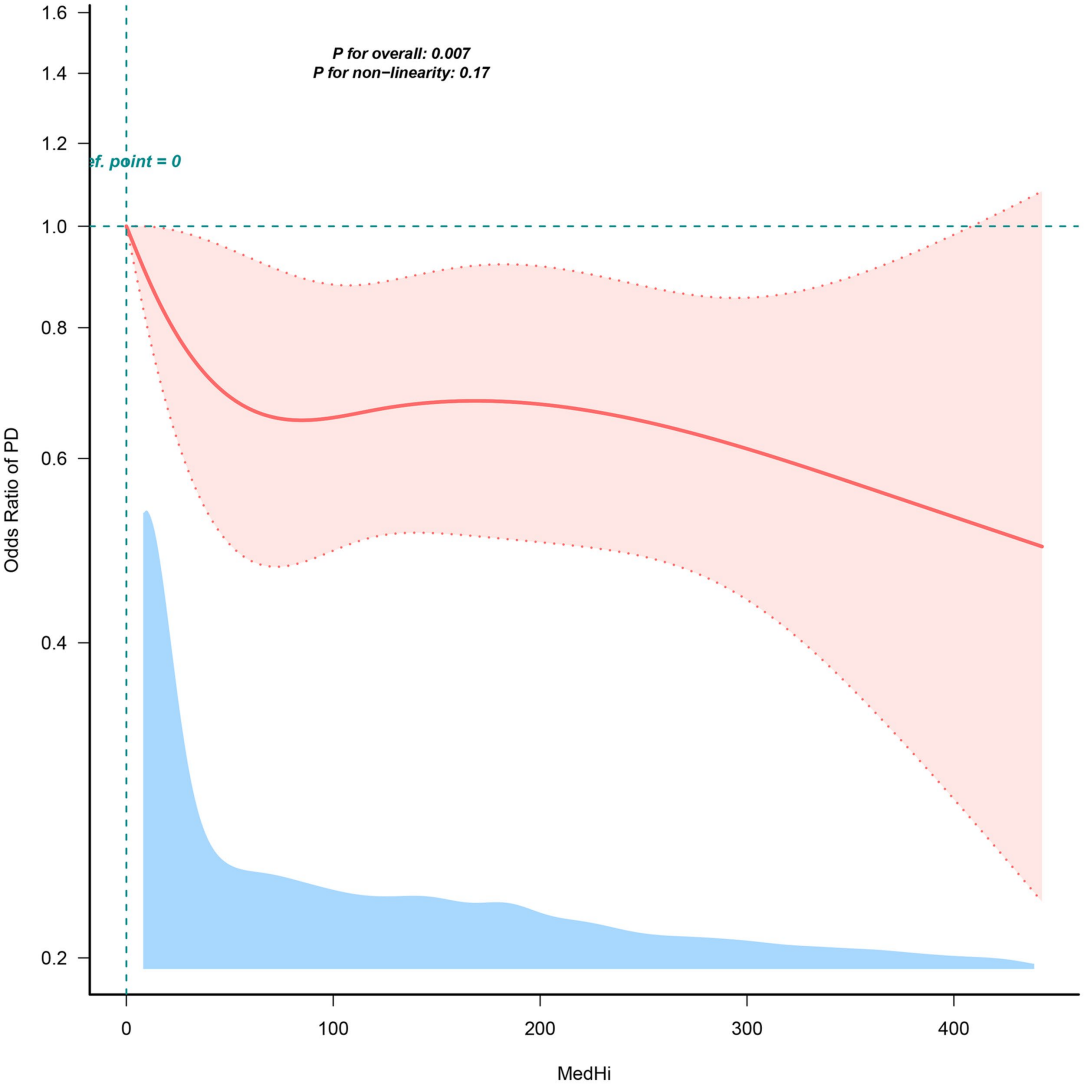
Restricted cubic spline fitting for the association between dietary live microbes’ intake and the prevalence of PD.

Table 2 presents the findings from logistic regression analyses examining the association between varying levels of dietary live microbe intake and the prevalence of PD. Upon accounting for all potential confounders, the analysis reveals a substantial decrease in the prevalence of PD among subjects with high (OR = 0.52, 95% CI: 0.37–0.74) and medium (OR = 0.73, 95% CI: 0.57–0.93) intake, compared to those with a low intake. Additionally, subjects were stratified into three groups based on MedHi intake to quantify the dietary live microbe intake. In the totally adjusted model, the prevalence of PD was found to be 31% lower in the G2 group (OR = 0.69, 95% CI: 0.52–0.90) and 36% lower in the G3 group (OR = 0.64, 95% CI: 0.48–0.85), compared to the G1 group. RCS analyses further illustrated a significant negative linear association between dietary live microbe intake and the prevalence of PD (Figure 3).

**Table 2:** Association between dietary live microbe intake and prevalence of PD.

Subgroups	Model 1		Model 2		Model 3	
	OR (95%CI)	*P*-value	OR (95%CI)	*P*-value	OR (95%CI)	*P*-value
Per100 unit increase	0.92 (0.85 ~ 0.99)	0.032	0.90 (0.83 ~ 0.98)	0.012	0.92 (0.84 ~ 0.99)	0.033
Category of MedHi^a^						
Low	1(Ref)		1(Ref)		1(Ref)	
Medium	0.74 (0.58 ~ 0.94)	0.015	0.69 (0.54 ~ 0.88)	0.003	0.73 (0.57 ~ 0.93)	0.010
High	0.54 (0.38 ~ 0.76)	<0.001	0.49 (0.34 ~ 0.69)	<0.001	0.52 (0.37 ~ 0.74)	<0.001
*P* for trend	0.73 (0.63 ~ 0.86)	<0.001	0.69 (0.59 ~ 0.82)	<0.001	0.72 (0.61 ~ 0.85)	<0.001
Category of MedHi^b^						
G1	1(Ref)		1(Ref)		1(Ref)	
G2	0.70 (0.54 ~ 0.92)		0.65 (0.50 ~ 0.86)	0.002	0.69 (0.52 ~ 0.90)	0.007
G3	0.65 (0.50 ~ 0.86)	<0.001	0.60 (0.46 ~ 0.79)	<0.001	0.64 (0.48 ~ 0.85)	0.002
*P* for trend	0.80 (0.70 ~ 0.92)	<0.001	0.77 (0.67 ~ 0.88)	<0.001	0.79 (0.69 ~ 0.91)	0.001

Model 1: None covariates were adjusted.

Model 2: gender, race and age were adjusted.

Model 3, gender, age, race, BMI, physical activities, diabetes, education level, hypertension, smoking status, CHD, stroke, cancer, PIR, hyperlipidemia, marital status, drinking status, total energy intake were adjusted.

^a^Participants were also classified into three different groups considering the general intake of foods with varying contents of microbe: low (all foods consumed were Low); moderate (any foods consumed were Medium but not High); and high (any foods consumed were High).

^b^Participants were categorized into three groups based on the MedHi consumption to quantify the ingestion of live microbes: G1, consumers without intakes of any MedHi food; G2, those with intakes of MedHi food above zero but below the median level for consumers; G3, those with intakes of MedHi food above the median level for consumers.

### Stratified analyses

**Figure 4:**
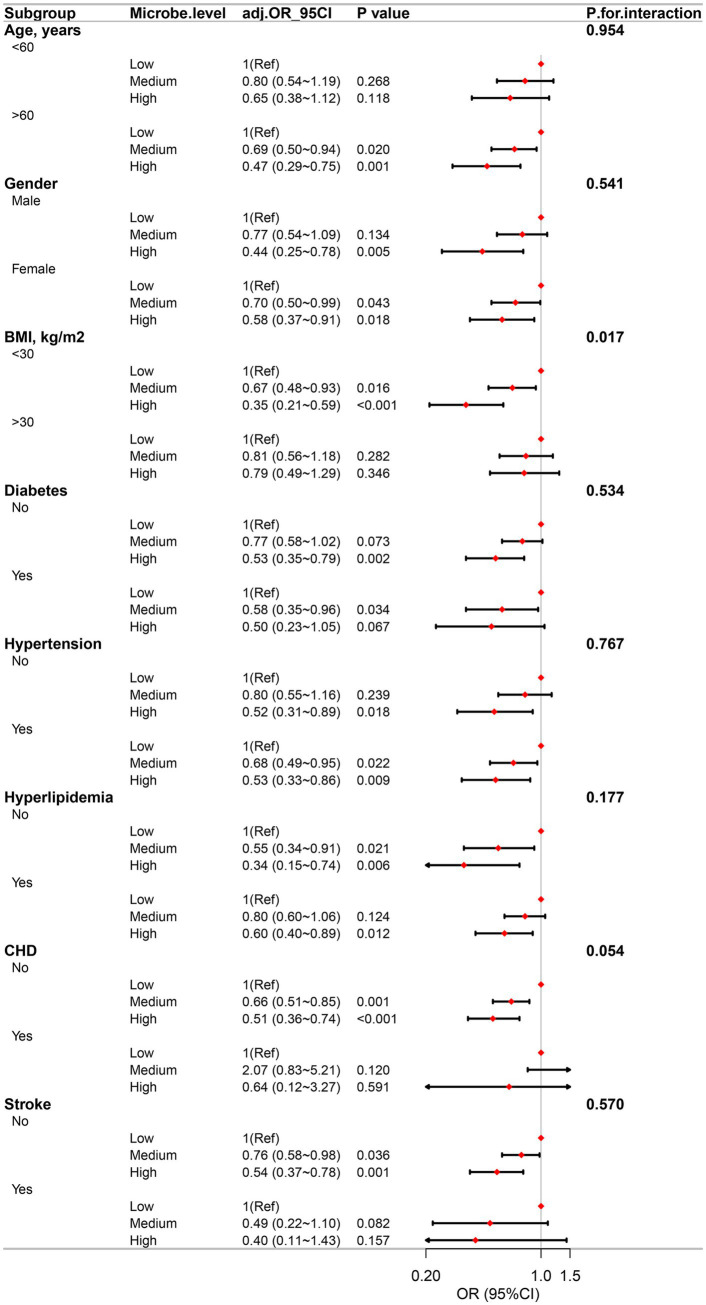
Association between high, medium and low dietary live microbes’ intake and the prevalence of PD in various subgroups.

**Figure 5:**
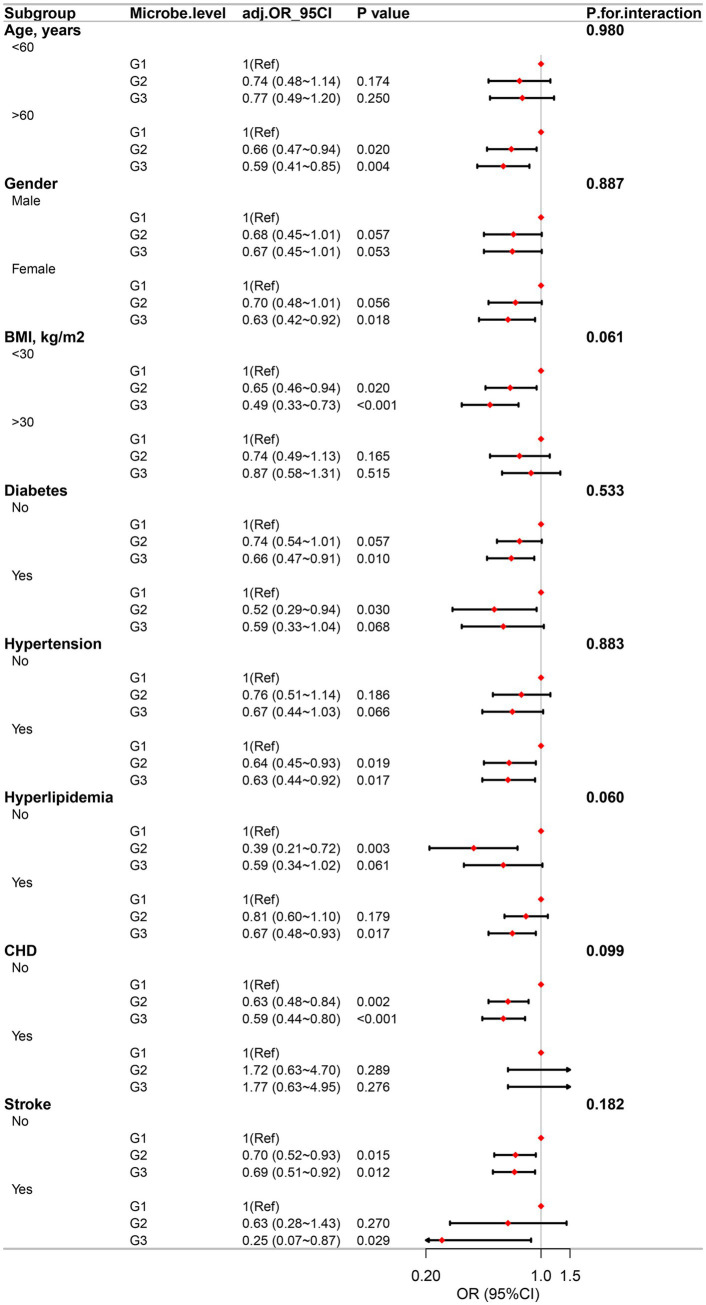
Association between G1, G2, and G3 dietary live microbes’ intake and the prevalence of PD in various subgroups.

To assess the stability of the association between dietary live microbe intake and PD, subgroup analyses and interaction tests were performed across various factors, including gender, age, and a history of diabetes, CHD, hypertension, hyperlipidemia, and stroke. These analyses did not reveal any significant association among these variables. Notably, the inverse association between a high dietary live microbe intake and the prevalence of PD was more pronounced in subjects without obesity, as illustrated in Figures 4, 5.

### Sensitivity analyses

To mitigate the potential confounding effects of non-dietary prebiotic and probiotic use, adjustments were incorporated into models (see Table 3). The analysis indicated a substantially lower prevalence of PD in the high intake group compared to the low intake group (OR = 0.85, 95% CI: 0.76–0.94). Moreover, the prevalence of PD was 47% lower in the G3 group than in the G1 group (OR = 0.53, 95% CI: 0.37–0.75).

**Table 3:** Association between dietary live microbe intake and prevalence of PD.

Subgroups	Model 1		Model 2		Model 3	
	OR (95%CI)	*P*-value	OR (95%CI)	*P*-value	OR (95%CI)	*P*-value
Per100 unit increase	0.92 (0.85 ~ 0.99)	0.032	0.92 (0.84 ~ 0.99)	0.033	0.85 (0.76 ~ 0.94)	0.002
Category of MedHi^a^						
Low	1(Ref)		1(Ref)		1(Ref)	
Medium	0.74 (0.58 ~ 0.94)	0.015	0.73 (0.57 ~ 0.93)	0.010	0.69 (0.51 ~ 0.93)	0.016
High	0.54 (0.38 ~ 0.76)	<0.001	0.52 (0.37 ~ 0.74)	<0.001	0.41 (0.26 ~ 0.63)	<0.001
*P* for trend	0.73 (0.63 ~ 0.86)	<0.001	0.72 (0.61 ~ 0.85)	<0.001	0.65 (0.53 ~ 0.8)	<0.001
Category of MedHi^b^						
G1	1(Ref)		1(Ref)		1(Ref)	
G2	0.70 (0.54 ~ 0.92)		0.69 (0.52 ~ 0.90)	0.007	0.68 (0.49 ~ 0.95)	0.022
G3	0.65 (0.50 ~ 0.86)	<0.001	0.64 (0.48 ~ 0.85)	0.002	0.53 (0.37 ~ 0.75)	<0.001
*P* for trend	0.80 (0.70 ~ 0.92)	<0.001	0.79 (0.69 ~ 0.91)	0.001	0.72 (0.61 ~ 0.86)	<0.001

Model 1: none covariates were adjusted.

Model 2: gender, age, race, BMI, physical activities, diabetes, education level, hypertension, smoking status, CHD, stroke, cancer, PIR, hyperlipidemia, marital status, drinking status, total energy intake, non-food prebiotic use (yes or no), and non-food probiotic use (yes or no) were adjusted.

Model 3: model 2+ non-food prebiotic use (yes or no), and non-food probiotic use (yes or no) were adjusted.

^a^Participants were also classified into three different groups considering the general intake of foods with varying contents of microbe: low (all foods consumed were Low); moderate (any foods consumed were Medium but not High); and high (any foods consumed were High).

^b^Participants were categorized into three groups based on the MedHi consumption to quantify the ingestion of live microbes: G1, consumers without intakes of any MedHi food; G2, those with intakes of MedHi food above zero but below the median level for consumers; G3, those with intakes of MedHi food above the median level for consume.

## Discussion

In this study, the data were gathered from a sample of 26,033 adults. After comprehensive analysis of the data, it was revealed that within the model adjusted for all confounding variables, the prevalence of PD decreased by 48 and 27% in the high and medium dietary live microbe intake groups, respectively, compared to the low intake group. RCS analyses demonstrated a linear and inverse association between dietary live microbe intake and the prevalence of PD, which was particularly pronounced among subjects without obesity. Sensitivity analyses further confirmed the reliability and consistency of these findings.

Numerous scholars have explored the impact of dietary live microbe intake on human health. Hill et al. (38) established an association between diet abundant in dietary live microbes and reductions in blood glucose levels, systolic blood pressure, inflammatory markers, and TG levels. Similarly, Han and Wang (39) reported that a higher dietary live microbe intake could potentially lower the risk of cardiovascular diseases. In addition, Wang et al. (32) found an inverse association between dietary live microbe intake and the incidence of depression. Moreover, those regularly consuming foods rich in dietary live microbes tended to display the improved cognitive performance (40). Numerous studies also have explored the potential benefits of probiotic supplements. Tamtaji et al. (41) proposed that an increased intake of prebiotics and probiotics may enhance gut flora homeostasis and reduce motor severity ratings in subjects with PD. Jin et al. (42) supported the hypothesis that prebiotics and probiotics may alleviate constipation symptoms in this population, through an analysis on 11 randomized controlled trials involving 765 PD patients. A recent meta-analysis conducted in 2023 suggested that probiotics may alleviate the depression and both motor and non-motor symptoms in subjects with PD (43). Similarly, it was the first time to find that subjects with a higher dietary live microbe intake may exhibit a reduced prevalence of PD.

The negative association between dietary live microbe intake and PD is more pronounced in non-obese individuals, which may be attributed to several key factors. Firstly, research indicates that obesity is associated with reduced microbiota diversity, characterized by an increased abundance of Firmicutes and a decreased abundance of Bacteroidetes (44). This imbalance may hinder the colonization and beneficial effects of dietary live microbes (45), whereas the diverse microbiota in non-obese individuals offers a more favorable environment for these microbes. Secondly, non-obese individuals experience lower levels of chronic inflammation compared to obese individuals, who often have elevated pro-inflammatory cytokines (46). The anti-inflammatory properties of dietary live microbes can be diminished in the presence of high baseline inflammation, which may reduce their protective effects against PD. Additionally, non-obese individuals typically demonstrate a more favorable metabolic profile, including better insulin sensitivity (47). In contrast, obesity is associated with metabolic disturbances that can increase oxidative stress and inflammation (48), potentially weakening the protective effects of dietary live microbes. Lastly, dietary patterns exhibit significant differences between the two groups. Obese individuals typically consume high-fat, high-sugar diets that are low in fiber and dietary live microbes, which exacerbate gut dysbiosis and diminish the benefits of dietary live microbes. On the contrary, non-obese individuals tend to have more balanced diets that are rich in fermented foods, thereby enhancing their overall health benefits (49, 50).

In this study, a significant correlation between the intake of dietary live microbes and a reduced risk of PD was identified. This correlation may be influenced by various metabolic and biological mechanisms. Firstly, dietary live microbes can modulate gut microbiota, thereby promoting the proliferation of beneficial bacterial genera such as *Bifidobacterium and lactobacilli* (51). These beneficial bacteria are known to produce short-chain fatty acids (SCFAs), including propionate, acetate, and butyrate, possessing neuroprotective properties and anti-inflammatory (52), which can strengthen the intestinal barrier, reducing microbial translocation and systemic inflammation which are key factors in the progression of PD (53). Additionally, dietary live microbe intakes can influence the immune system by stimulating the production of anti-inflammatory cytokines and enhancing the function of regulatory T cells (54), helping to mitigate the chronic low-grade inflammation often observed in PD patients. Furthermore, these microbes may also exert antioxidant effects, reducing oxidative stress, a significant contributor to neuronal damage in PD (55, 56). Dietary live microbe intakes can offer a promising non-pharmacological approach by improving gut health, modulating inflammation, and reducing oxidative stress, to potentially lower the risk of PD.

To the best of our knowledge, this is the first study to explore the correlation between dietary microbe intake and the prevalence of PD. This study possesses several notable strengths. Firstly, the data adopted in this study were sourced from NHANES, a nationally representative database, with data collection and compilation conducted by professionals. Secondly, the study assessed subjects’ dietary live microbe intake through their self-reported food intake. Microbe intake was quantified by categorizing subjects into high, medium, and low intake groups based on microbial content, as well as into G3, G2, and G1 groups according to MedHi intake. Thirdly, the overall microbial content of all diets has been estimated by analyzing the data from 24-h dietary recall assessments, rather than concentrating exclusively on the intake of probiotics and the supplements.

However, it is essential to recognize several limitations inherent in this study. Firstly, the database employed in this study defined PD based on the anti-Parkinsonian medication rather than a clinical diagnosis by healthcare professionals, leading to potential biases. Specifically, subjects with mild PD symptoms who do not require medication might have been included in the control group, while subjects with other neurological disorders prescribed drugs on PD might have been misclassified into the PD group. Secondly, the approach endorsed by the International Society for the Science of Probiotics and Prebiotics for classifying the dietary live microbe intake was adopted in order to gauge the microbial content. Although these classification principles are primarily based on statistical values (9, 57–59) and authoritative reviews from previous literature (30), they predominantly rely on indirect assessments rather than direct microbial testing. Furthermore, these methods do not take into account potential variations in microbial content resulting from factors such as food preparation, storage, and processing. This oversight may contribute to misclassifications in the assessment of dietary live microbe intake levels. However, direct assessment requires a long time and huge expenditure, which limits its application. Thirdly, due to the varied dietary habits among individuals with different dietary patterns, further research is needed to explore the association between dietary live microbe intake from different food sources and the prevalence of PD. Fourthly, given the cross-sectional design of this study, it is crucial to acknowledge that causal inferences cannot be conducted. Fifthly, the potential impact of unidentified confounding variables may limit the interpretability of these findings, particularly concerning the consumption of other specific food groups that are rich in live microorganisms. Finally, the study was unable to determine whether the observed health benefits were attributable to the food itself or to the presence of live bacteria within the food, thereby limiting the ability to ascertain the true impact on the prevalence of PD.

## Conclusion

To sum up, this study indicates that a higher dietary live microbe intake associates with a lower prevalence of PD. Given the increasing prevalence of PD and the possible neuroprotective effects of dietary live microbes, the results of this study have important ramifications for public health initiatives and clinical approaches.

## Data Availability

Publicly available datasets were analyzed in this study. This data can be found here: NHANES, https://wwwn.cdc.gov/nchs/nhanes.
