# Supplementary material for: Dietary live microbe intake and its association with Parkinson’s disease in American adults: an NHANES analysis (1999–2018)
Source: Front Nutr. 2025 May 29;12:1606922. doi: 10.3389/fnut.2025.1606922 (PMC12158673; doi:10.3389/fnut.2025.1606922)
Supplement: Supplementary file 1 [file Table_1.docx]

Table S1 Clinical characteristics of participants by dietary live microbe groups.

| **Characteristics** |  | **Category of MedHi** |  | **P-value** |
| --- | --- | --- | --- | --- |
|  | **Low** | **Medium** | **High** |  |
| Age, year | 59.6 ± 12.5 | 60.7 ± 12.6 | 59.2 ± 12.5 | <0.001 |
| Gender, n (%) |  |  |  | <0.001 |
| Male | 4806 (53.5) | 5748 (48.7) | 2287 (43.7) |  |
| Female | 4184 (46.5) | 6060 (51.3) | 2948 (56.3) |  |
| PIR | 2.32 ± 1.55 | 2.75 ± 1.61 | 3.12 ± 1.64 | < 0.001 |
| BMI, kg/m^2^ | 29.6 ± 6.7 | 29.2 ± 6.3 | 29.1 ± 6.6 | <0.001 |
| Race, n (%) |  |  |  | <0.001 |
| Mexican American | 1308 (14.5) | 2239 (19) | 698 (13.3) |  |
| Other Hispanic | 719 (8) | 867 (7.3) | 459 (8.8) |  |
| Non-Hispanic  White | 3818 (42.5) | 5895 (49.9) | 3103 (59.3) |  |
| Non-Hispanic Black | 2608 (29) | 2085 (17.7) | 635 (12.1) |  |
| Other Race | 537 (6) | 722 (6.1) | 340 (6.5) |  |
| Physical activity, n (%) |  |  |  | 0.231 |
| Low | 7533 (83.8) | 10060 (85.2) | 4460 (85.2) |  |
| High | 1457 (16.2) | 1748 (14.8) | 775 (14.8) |  |
| Marital group, n (%) |  |  |  | <0.001 |
| Married or living with partner | 5569 (61.9) | 8073 (68.4) | 3695 (70.6) |  |
| Widowed or divorced | 2589 (28.8) | 2977 (25.2) | 1214 (23.2) |  |
| Never married | 832 (9.3) | 758 (6.4) | 326 (6.2) |  |
| Education level, n (%) |  |  |  | <0.001 |
| Less than high school | 3248 (36.1) | 3418 (28.9) | 1007 (19.2) |  |
| High school or above | 5742 (63.9) | 8390 (71.1) | 4228 (80.8) |  |
| Smoking status, n (%) |  |  |  | <0.001 |
| Current or ever | 4951 (55.1) | 5735 (48.6) | 2437 (46.6) |  |
| Never | 4039 (44.9) | 6073 (51.4) | 2798 (53.4) |  |
| Drinking status, n (%) |  |  |  | <0.001 |
| Current or ever | 6044 (67.2) | 7972 (67.5) | 3765 (71.9) |  |
| Never | 2946 (32.8) | 3836 (32.5) | 1470 (28.1) |  |
| Diabetes, n (%) | 1824 (20.3) | 2352 (19.9) | 845 (16.1) | <0.001 |
| Hypertension, n (%) | 4400 (48.9) | 5493 (46.5) | 2255 (43.1) | <0.001 |
| Hyperlipidemia, n (%) | 6845 (76.1) | 9195 (77.9) | 3977 (76) | 0.003 |
| CHD, n (%) | 565 (6.3) | 781 (6.6) | 287 (5.5) | 0.019 |
| Stroke, n (%) | 575 (6.4) | 552 (4.7) | 190 (3.6) | <0.001 |
| Cancer, n (%) | 1052 (11.7) | 1629 (13.8) | 805 (15.4) | <0.001 |
| Total energy intake, kcal | 1895.5 ± 922.6 | 1979.8 ± 889.7 | 2108.3 ± 919.1 | <0.001 |
| PD, n (%) | 137 (1.5) | 134 (1.1) | 43 (0.8) | <0.001 |

Values are mean±SD, median (IQR) or number (%). BMI, body mass index; PIR, poverty-income ratio; CHD, coronary heart disease;

^a^Participants were also classified into three different groups considering the general intake of foods with varying contents of microbe: low (all foods consumed were Low); moderate (any foods consumed were Medium but not High); and high (any foods consumed were High)

^b^Participants were categorized into three groups based on the MedHi consumption to quantify the ingestion of live microbes: G1, consumers without intakes of any MedHi food; G2, those with intakes of MedHi food above zero but below the median level for consumers; G3, those with intakes of MedHi food above the median level for consumers

Table S2 Clinical characteristics of participants by dietary live microbe groups.

| **Characteristics** |  | **Category of MedHi** |  | **P-value** |
| --- | --- | --- | --- | --- |
|  | **G1** | **G2** | **G3** |  |
| Age, year | 59.6 ± 12.5 | 60.1 ± 12.8 | 60.3 ± 12.4 | 0.001 |
| Gender, n (%) |  |  |  | <0.001 |
| Male | 4806 (53.5) | 4060 (47.9) | 3975 (46.4) |  |
| Female | 4184 (46.5) | 4412 (52.1) | 4596 (53.6) |  |
| PIR | 2.32 ± 1.55 | 2.73 ± 1.61 | 3.00 ± 1.64 | < 0.001 |
| BMI, kg/m^2^ | 29.6 ± 6.7 | 29.2 ± 6.4 | 29.0 ± 6.4 | <0.001 |
| Race, n (%) |  |  |  | <0.001 |
| Mexican American | 1308 (14.5) | 1532 (18.1) | 1405 (16.4) |  |
| Other Hispanic | 719 (8) | 661 (7.8) | 665 (7.8) |  |
| Non-Hispanic  White | 3818 (42.5) | 4335 (51.2) | 4663 (54.4) |  |
| Non-Hispanic Black | 2608 (29) | 1477 (17.4) | 1243 (14.5) |  |
| Other Race | 537 (6) | 467 (5.5) | 595 (6.9) |  |
| Physical activity, n (%) |  |  |  | 0.171 |
| Low | 7533 (83.8) | 7185 (84.8) | 7328 (85.5) |  |
| High | 1457 (16.2) | 1287 (15.2) | 1243 (14.5) |  |
| Marital group, n (%) |  |  |  | <0.001 |
| Married or living with partner | 5569 (61.9) | 5823 (68.7) | 5945 (69.4) |  |
| Widowed or divorced | 2589 (28.8) | 2117 (25) | 2074 (24.2) |  |
| Never married | 832 (9.3) | 532 (6.3) | 552 (6.4) |  |
| Education level, n (%) |  |  |  | <0.001 |
| Less than high school | 3248 (36.1) | 2432 (28.7) | 1993 (23.3) |  |
| High school or above | 5742 (63.9) | 6040 (71.3) | 6578 (76.7) |  |
| Smoking status, n (%) |  |  |  | <0.001 |
| Current or ever | 4951 (55.1) | 4239 (50) | 3933 (45.9) |  |
| Never | 4039 (44.9) | 4233 (50) | 4638 (54.1) |  |
| Drinking status, n (%) |  |  |  | 0.026 |
| Current or ever | 6044 (67.2) | 5840 (68.9) | 5897 (68.8) |  |
| Never | 2946 (32.8) | 2632 (31.1) | 2674 (31.2) |  |
| Diabetes, n (%) | 1824 (20.3) | 1607 (19) | 1590 (18.6) | 0.009 |
| Hypertension, n (%) | 4400 (48.9) | 3942 (46.5) | 3806 (44.4) | <0.001 |
| Hyperlipidemia, n (%) | 6845 (76.1) | 6562 (77.5) | 6610 (77.1) | 0.099 |
| CHD, n (%) | 565 (6.3) | 525 (6.2) | 543 (6.3) | 0.931 |
| Stroke, n (%) | 575 (6.4) | 414 (4.9) | 328 (3.8) | <0.001 |
| Cancer, n (%) | 1052 (11.7) | 1204 (14.2) | 1230 (14.4) | <0.001 |
| Total energy intake, kcal | 1895.5 ± 922.6 | 1960.1 ± 890.6 | 2077.8 ± 906.9 | <0.001 |
| PD, n (%) | 137 (1.5) | 91 (1.1) | 86 (1.0) | 0.003 |

Values are mean±SD, median (IQR) or number (%). BMI, body mass index; PIR, poverty-income ratio; CHD, coronary heart disease;

^a^Participants were also classified into three different groups considering the general intake of foods with varying contents of microbe: low (all foods consumed were Low); moderate (any foods consumed were Medium but not High); and high (any foods consumed were High)

^b^Participants were categorized into three groups based on the MedHi consumption to quantify the ingestion of live microbes: G1, consumers without intakes of any MedHi food; G2, those with intakes of MedHi food above zero but below the median level for consumers; G3, those with intakes of MedHi food above the median level for consumers
